# BMI Specific Complications Following Implant-Based Breast Reconstruction after Mastectomy

**DOI:** 10.3390/jcm10235665

**Published:** 2021-11-30

**Authors:** Helena Sophie Leitner, Reinhard Pauzenberger, Ines Ana Ederer, Christine Radtke, Stefan Hacker

**Affiliations:** 1 Department of Plastic, Reconstructive and Aesthetic Surgery, Medical University of Vienna, 1090 Vienna, Austria; helenaleitner@gmx.at (H.S.L.); reinhard.pauzenberger@gmx.at (R.P.); christine.radtke@meduniwien.ac.at (C.R.); 2 Privatordination Dr. Pauzenberger, 4861 Schörfling am Attersee, Austria; 3 Department of Plastic and Aesthetic, Reconstructive and Hand Surgery, AGAPLESION Markus Hospital, 60431 Frankfurt, Germany; ines.ederer@hotmail.com; 4 Department of Plastic, Aesthetic and Reconstructive Surgery, State Hospital Wiener Neustadt, 2700 Wiener Neustadt, Austria

**Keywords:** breast reconstruction, body mass index, implants, mastectomy, complications, obesity, overweight, expander

## Abstract

Background: Breast reconstruction has a positive impact on body image and quality of life for women after experiencing the physically and psychologically demanding process of mastectomy. Previous studies have presented body mass index (BMI) as a predictor for postoperative complications after breast reconstruction, however, study results vary. This retrospective study aimed to investigate the impact of patients’ BMI on postoperative complications following implant-based breast reconstruction. Methods: All implant-based breast reconstructions performed at the Department of Plastic, Reconstructive and Aesthetic Surgery at the Medical University of Vienna from January 2001 to March 2018 were evaluated. A total of 196 reconstructed breasts among 134 patients met eligibility criteria. Demographic data, surgical techniques, as well as major and minor complications within a one-year follow-up period were analyzed. Results: Patients’ BMI did not show a significant impact on complication rates. The overall incidence of postoperative complications was 30.5% (40/131) of which 17.6% required reoperation. Impaired wound healing (18.3%), seroma (6.1%), hematoma (4.6%), capsular contraction (4.6%) and infection (3.8%) were the most common complications. Conclusion: In our study cohort, BMI was not associated with a significantly higher risk of complications. However, postoperative complications significantly increased with a longer operative time and resulted in an extended length of hospital stay.

## 1. Introduction

With almost 2.3 million cases in 2020, breast cancer is the most common type of cancer in women worldwide [1]. Due to evolving technology and modalities in diagnosis and treatment, the survival rate has increased over the past decade [2]. Non-invasive therapies (hormone therapy, radiotherapy, chemotherapy) have become widely available; however, surgical treatment is still desirable in most cases [3,4]. Breast-conserving surgery is preferred by most patients and physicians alike. In cases of prophylactic treatment, inflammatory breast cancer, non-resectable tumors or when radiotherapy following breast-conserving surgery is contraindicated or not wished by the patient, mastectomy is indicated [3,5,6,7,8]. Even though mastectomy is an effective treatment option, it may be a demanding process for the patient—both physically and psychologically. With a wide variety of different breast reconstruction techniques—including implant-based and autologous—it is an excellent option to improve patients’ body image and quality of life [9,10,11]. As for these surgeries, risk stratification should be individualized for every patient depending on surgical technique, implant characteristics and patients’ demographic data. For example, smoking cessation is highly advised preoperatively due to the increased risk of complications and reconstructive failure in smokers undergoing expander/implant breast reconstruction [12,13,14,15].

Opting for ideal patient selection, obesity as a prognostic factor for surgery has become a well-researched topic. The prevalence of obesity is constantly increasing and affects most medical disciplines [16]. It was repeatedly demonstrated that obesity is associated with a variety of comorbidities affecting multiple organ systems such as diabetes mellitus, arterial hypertension, hyperlipidemia, stroke and coronary heart disease. In addition, being overweight was shown to be a risk factor for malignant diseases including breast cancer [17,18,19].

Previous studies have addressed the effect of obesity on outcomes following breast surgery; however, available data are inconclusive as the reported results vary [13,15,19,20,21,22,23]. In this study, we aimed to further investigate the influence of BMI on postoperative complications following implant-based breast reconstruction in order to improve patient selection and mitigate complications and adverse outcomes.

## 2. Materials and Methods

### 2.1. Study Design

We retrospectively analyzed all patients who underwent implant-based breast reconstruction following mastectomy for prophylactic or oncological reasons between January 2001 and March 2018. Breast reconstructions were conducted immediately or delayed in a one or two-stage procedure with either implants or expanders in patients with a limited skin envelope after mastectomy, respectively. Patients who underwent combined autologous assisted implant reconstruction—e.g., with a latissimus dorsi flap—were also included. Exclusion criteria were patients under the age of 18 and secondary reconstructions after previous failure. In case of missing key variables—such as BMI—patients were excluded. A total of 134 patients met the inclusion criteria. Three patients were lost to follow-up and were not included in the statistical analysis.

Data on age, weight, height, BMI, comorbidities (such as diabetes mellitus, arterial hypertension), smoking status, implant characteristics, implant positioning, the additional use of a latissimus dorsi flap, timing of reconstruction, surgery time, length of stay and duration of follow-up were registered in a computerized database for each patient.

According to the WHO classification, BMI was categorized into six groups: underweight (<18.5), normal weight (18.5–24.9), pre-obesity (25.0–29.9), obesity class I (30.0–34.9), obesity class II (35.0–39.9) and obesity class III (≥40) [22]. Considering the low number of obese patients in our study, obesity class I-III were summarized (≥30) and statistical analysis was performed by comparing obese and non-obese patients.

Complications were recorded during a 1 year follow-up period. These included wound-healing disorder, hematoma, seroma, infection, implant rupture and capsular fibrosis. Depending on the treatment method, further differentiation was made into minor (conservative treatment) and major complications (necessity of revision surgery).

### 2.2. Statistical Analysis

Descriptive statistics were used to describe patients’ demographics, surgical details and postoperative complications. Data are reported by number (n) or percentage (%). For continuously coded variables, means and standard deviations were computed, while proportions were used to describe categorical variables. Group-specific differences of surgical variables or patients’ demographics were analyzed with the chi-square test and t-test or the nonparametric equivalents when normality distribution was not given.

All analyses were conducted with IBM SPSS Statistics 27 (IBM SPSS Statistics for iOS, Version 27.0.1.0, Armonk, NY, USA). Statistical testing was performed two-sided with the significance level set at *p* < 0.05.

## 3. Results

### 3.1. Patient Characteristics

Over the study period, a total of 134 patients were identified. The mean age was 47.0 ± 10.6 years (range: 27–76). In 62 patients, reconstruction was performed bilaterally; in 44 patients it affected the right and in 28 the left side. A total of 196 implants, including 128 expanders were used. The mean implant volume was 320.5 ± 101.9 cm³ (range: 120–685). The implant position was submuscular in the majority of cases (*n* = 126, 94%); in eight (6%) cases, prepectoral positioning was performed. In 11 patients a latissimus-dorsi-flap was used together with an implant during the same procedure.

### 3.2. Complications

In total, 51 postoperative complications occurred in 40 patients (30.5%) including 17.6% major complications and 13.0% minor complications. Complication rates were higher in the case of autologous assisted breast reconstruction (45.5%) than in the implant-based reconstructions (29.1%).

Table 1 demonstrates all complications, with impaired wound healing being the most common (18.3%, *n* = 24) which also includes skin (12.2%, *n* = 16) and fat necrosis (3.8%, *n* = 5). Among those, 50% (*n* = 12) required revision surgery. Further indications for a surgical revision were hematoma, seroma, capsular fibrosis, implant rupture and infections.

All cases of capsular fibrosis affected patients who underwent immediate reconstruction. In three patients, capsular fibrosis occurred following impaired wound healing. Revision surgery was performed after a mean of 6.4 months. Capsular fibrosis was less common when using expanders (3.4%) compared to standard silicone implants (7.1%). Both cases of implant rupture happened in patients who received expanders.

Infections have been reported exclusively after immediate reconstruction and all cases led to the loss of the implant. Overall, in 11.5% of the cases, complications led to the loss of the implant, and in 4.6% a new implant could be inserted during the same procedure.

### 3.3. Body Mass Index (BMI)

The mean BMI in the study cohort was 23.2 ± 4.4 (min. 16.1, max. 43.6) kg/m^2^. Categorization of BMI according to the WHO classification is further shown in Table 2. Almost one quarter of all patients (24.6%) were overweight (>25 kg/m^2^) including 5.9% obese.

**Figure 1:**
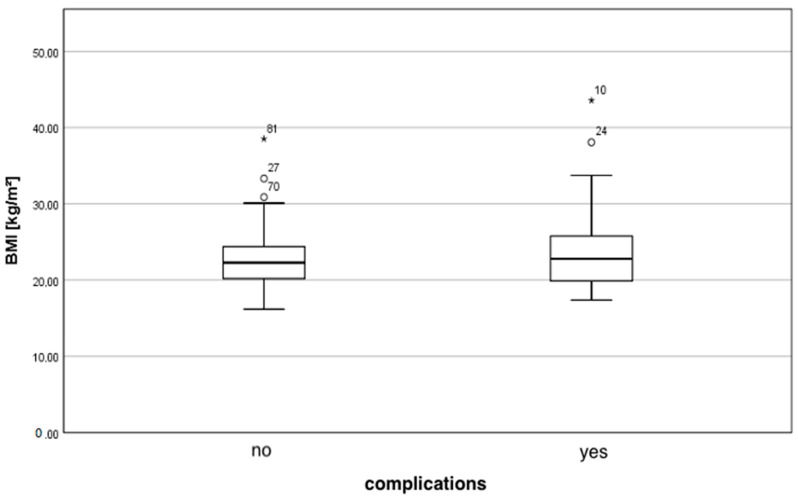
Distribution of the BMI depending on complications.

Figure 1 displays the distribution of BMI depending on the occurrence of postoperative complications following breast reconstruction. No significant difference between the mean BMI in the group with complications (23.9 ± 5.7 kg/m^2^) and without complications (22.8 ± 3.7 kg/m^2^) was found (*p* = 0.646).

Comparing the cohorts of underweight, normal weight and overweight patients, complications showed the highest incidence in the group of overweight patients (40.6%), followed by underweight patients (36.4%). Normal weight patients were less likely to have complications (26.1%) compared to both other groups; this, however, did not result in a statistically significant difference (*p* = 0.284).

Obese patients suffered from postoperative complications in 50% of cases. No significant difference in developing postoperative complications between obese and non-obese patients was found (major complications: *p* = 0.146, minor complications: *p* = 0.723).

As for patients who underwent autologous assisted implant-based reconstruction, BMI significantly differed from patients who experienced postoperative complications than those who had an uneventful follow-up (*p* = 0.028). Further details on the incidence of complications depending on BMI are shown in Appendix A Table A1.

### 3.4. Comorbidities and Other Risk Factors

In addition to BMI, other risk factors were evaluated. These included age > 60 years, arterial hypertension, diabetes mellitus and smoking. There was no significant association between one of these factors and the incidence of postoperative complications.

### 3.5. Surgical Details

Implant volume and implant positioning had no significant impact on the incidence of postoperative complications. Comparing the rate of complication using silicone implants (45.2%) and expanders (22.7%), the first mentioned had an increased risk of postoperative complications (*p* = 0.009). No significant difference was found in the complication rates of immediate or delayed breast reconstructions.

Prolonged surgery time was significantly associated with an increase in postoperative complications (*p* < 0.001). Patients who did not experience complications had a mean surgery time of 176.4 min, while patients suffering from postoperative complications had a mean surgery time of 229.4 min. The length of stay significantly increased in cases with postoperative complications (9.2 days ± 3.8 vs. 11.3 ± 4.6; *p* = 0.002).

## 4. Discussion

Considering the high prevalence of overweight and obesity in our society, the possible consequences for complications after breast reconstruction should be taken into consideration. Therefore, we analyzed the association of BMI on postoperative complications following implant-based breast reconstruction after mastectomy.

The results demonstrate that BMI had no significant impact on the incidence of postoperative complications in our study cohort. Obese patients showed a tendency towards a higher risk of complications, but this observance did not result in a significant difference from other groups. In cases of autologous assisted breast reconstruction, however, the BMI was significantly higher among patients suffering from postoperative complications. Due to the small number of patients in our study being obese, further investigations are warranted to draw a conclusion.

Comparing our results to other studies, differences vary [20,21,25]. Hanwright et al. [25] analyzed the differential effect of BMI on prosthetic versus autologous breast reconstruction in 12,986 patients. As for obese patients reconstructed by autologous tissue, the rate of reoperation (12.8% versus 9.1%), overall morbidity (18.0% versus 9.5%), surgical (12.7% versus 8.3%) and medical complications (9.0% versus 2.2%) were higher compared to tissue expander recipients. Compared to non-obese patients, overall morbidity significantly increased in obese patients across all forms of reconstruction.

Likewise, Fischer et al. [21] concluded that an increase in BMI was directly related to a higher likelihood of major surgical complications, implant and flap loss and wound complications including wound dehiscence and infections. Nguyen et al. [20] were able to demonstrate a 5.9 % increase in the odds of complications occurring by every unit increase in BMI. They concluded that by considering BMI rather than obesity, surgeons may be able to better predict the outcome. In this study, implants were positioned in a subpectoral plane. This stands in contrast to Gabriel et al. [26], who evaluated the effect of BMI on complications after prepectoral reconstruction and did not describe it as an independent predictor. The authors do not recommend using BMI alone for a risk estimation. In a further investigation, Gabriel et al. [27] compared dual-plane versus prepectoral breast reconstruction in high BMI patients. The prepectoral approach was associated with lower rates of seroma, surgical-site infection, capsular contracture and overall complications (25.8% versus 14.7%) and thus may be preferred in high-BMI patients.

Our data, however, did not show an association of BMI with postoperative complications. Therefore, we would not recommend considering BMI as an independent risk factor following implant-based breast reconstruction. Nevertheless, previous studies have shown the association of obesity with comorbidities such as hyperlipidemia, arterial hypertension and diabetes mellitus. These factors are associated with vascular pathologies, leading to circulatory disorders and surgical complications such as impaired wound healing [18,28,29,30]. Considering this aspect, a higher BMI may be seen as a warning sign for possible risk factors, but not as a risk factor itself.

Being underweight may increase the risk of postoperative complications in patients undergoing surgery as shown in previous studies [31,32]. Nevertheless, evidence is still missing regarding the risks of breast reconstruction in underweight patients. Weichman et al. [33] compared the quality of life after autologous breast reconstruction with prosthetic implant-based reconstruction in thin patients (BMI < 22 kg/m^2^). They showed higher patient satisfaction after autologous reconstruction, although leading to a higher frequency of secondary revision surgery. Thus, further studies are needed concerning implant-based breast reconstruction in thin and/or underweight patients.

In our study, underweight patients had an increased likelihood of complications compared to normal weight patients. This might be due to a limited skin envelope leading to more tension and a higher risk of skin necrosis [34,35]. However, our results are limited by the small number of patients, especially as far as underweight and obese patients are concerned. Further limitations of this study are related to its retrospective design. These include a certain inhomogeneity of performing surgeons within the observed study period. Moreover, the evolution and improvements in modern breast surgery and reconstruction over the study period may have influenced our results.

## 5. Conclusions

In this retrospective study, postoperative complications significantly increased with operative time and caused an extended length of stay. Using expanders resulted in significantly fewer complications than in the case of silicone implants. BMI, however, was not associated with a higher risk of postoperative complications.

## Figures and Tables

**Table 1:** Postoperative complications.

Complications	Major	Minor	Total (*n* = 131)
Impaired wound healing	12 (9.15%)	12 (9.15%)	24 (18.3%)
Hematoma	5 (3.8%)	1 (0.8%)	6 (4.6%)
Seroma	2 (1.5%)	6 (4.6%)	8 (6.1%)
Capsular fibrosis	6 (4.6%)	0	6 (4.6%)
Rupture	2 (1.5%)	0	2 (1.5%)
Infection	5 (3.8%)	0	5 (3.8%)

**Table 2:** BMI classification according to the WHO [24] and number of patients in each group.

BMI	Nutritional Status	Number of Patients
<8.5	Underweight	11 (8.2%)
18.5–24.9	Normal weight	90 (67.2%)
25.0–29.9	Pre-obesity	25 (18.7%)
30.0–34.9	Obesity class I	5 (3.7%)
35.0–39.9	Obesity class II	2 (1.5%)
≥40	Obesity class III	1 (0.7%)
	Total	134 (100 %)

## Appendix A

**Table A1:** Statistical results; M = mean, Md = median, SD = standard deviation.

Parameters	Complications		*p*
	Yes (*n* = 40; 30.5%)	No (*n* = 91; 69.5%)	
Implant type	(*n* = 39; 29.1%)	(*n* = 91; 69.5%)	0.009
Silicone	19 (47.5%)	23 (25.3%)	
Expander	20 (50.0%)	68 (74.7%)	
Age (y)			0.717
M ± SD	47.7 ± 10.7	46.5 ± 10.7	
Md (min—max)	47 (30–73)	46 (27–76)	
BMI (kg/m²)			0.646
M ± SD	23.9 ± 5.7	22.8 ± 3.7	
Md (min—max)	22.8 (17.4–43.6)	22.3 (16.1–38.5)	
Underweight	4 (10.0%)	7 (7.7%)	0.284
Normal weight	23 (57.5%)	65 (71.4%)	
Overweight	13 (32.5%)	19 (20.9%)	
	(*n* = 40; 30.5%)	(*n* = 91; 69.5%)	0.198
Obese	4 (10.0%)	4 (4.4%)	
Non-obese	36 (90.0%)	87 (95.6%)	
Comorbidities	(*n* = 21; 16.0%)	(*n* = 41; 31.3%)	
Arterial hypertension	7	13	0.638
Diabetes mellitus	2	2	0.585
Smokers	12	26	0.868
Timing of reconstruction	(*n* = 40; 30.5%)	(*n* = 91; 69.5%)	0.198
Immediate	33 (82.5%)	65 (71.4%)	
Delayed	7 (17.5%)	26 (28.6%)	
Surgery time (min)			<0.001
M ± SD	229.4 ± 78.5	176.4 ± 70.1	
Md (min-max)	235 (60–400)	180 (45–335)	
Implant volume (cm^3^)			0.984
M ± SD	325.8 ± 114.4	318.1 ± 96.4	
Md (min-max)	300 (120–685)	315 (120–685)	
Implant position	(*n* = 40; 30.5%)	(*n* = 91; 69.5%)	1
Submuscular	38 (95%)	86 (94.5%)	
Prepectoral	2 (5%)	5 (5.5%)	
LD-flap	(*n* = 40; 100%)	(*n* = 91; 100%)	0.309
With	5 (12.5%)	6 (6.6%)	
Without	35 (87.5%)	85 (93.4%)	
Length of stay			0.002
M ± SD	11.3 ± 4.6	9.2 ± 3.8	
Md (min-max)	10 (5–29)	9 (4–38)	

## References

[1] Sung H., Ferlay J., Siegel R.L., Laversanne M., Soerjomataram I., Jemal A., Bray F. (2021). Global Cancer Statistics 2020: GLOBOCAN Estimates of Incidence and Mortality Worldwide for 36 Cancers in 185 Countries. CA. Cancer J. Clin..

[2] Rojas K., Stuckey A. (2016). Breast Cancer Epidemiology and Risk Factors. Clin. Obstet. Gynecol..

[3] Hindle W. (2002). Breast cancer: Introduction. Clin. Obstet. Gynecol..

[4] Waks A.G., Winer E.P. (2019). Breast Cancer Treatment: A Review. JAMA.

[5] Brackstone M., Fletcher G.G., Dayes I.S., Madarnas Y., Sen Gupta S.K., Verma S., Eisen A., Gandhi S., Holloway C., Trudeau M. (2015). Locoregional therapy of locally advanced breast cancer: A clinical practice guideline. Curr. Oncol..

[6] Voogd A.C., Nielsen M., Peterse J.L., Blichert-Toft M., Bartelink H., Overgaard M., van Tienhoven G., Andersen K.W., Sylvester R.J., van Dongen J.A. (2001). Differences in risk factors for local and distant recurrence after breast-conserving therapy or mastectomy for stage I and II breast cancer: Pooled results of two large European randomized trials. J. Clin. Oncol..

[7] Fisher B., Anderson S. (1994). Conservative surgery for the management of invasive and noninvasive carcinoma of the breast: NSABP trials. National Surgical Adjuvant Breast and Bowel Project. World J. Surg..

[8] New Zealand Guidelines Group (2009). Management of Early Breast Cancer—Evidence-Based Best Practice Guideline.

[9] Serletti J.M., Fosnot J., Nelson J.A., Disa J.J., Bucky L.P. (2011). Breast reconstruction after breast cancer. Plast. Reconstr. Surg..

[10] Toyserkani N.M., Jørgensen M.G., Tabatabaeifar S., Damsgaard T., Sørensen J.A. (2020). Autologous versus implant-based breast reconstruction: A systematic review and meta-analysis of Breast-Q patient-reported outcomes. J. Plast. Reconstr. Aesthet. Surg..

[11] Guyomard V., Leinster S., Wilkinson M. (2007). Systematic review of studies of patients’ satisfaction with breast reconstruction after mastectomy. Breast.

[12] Goodwin S.J., McCarthy C.M., Pusic A.L., Bui D., Howard M., Disa J.J., Cordeiro P.G., Mehrara B.J. (2005). Complications in smokers after postmastectomy tissue expander/implant breast reconstruction. Ann. Plast. Surg..

[13] McCarthy C.M., Mehrara B.J., Riedel E., Davidge K., Hinson A., Disa J.J., Cordeiro P.G., Pusic A.L. (2008). Predicting complications following expander/implant breast reconstruction: An outcomes analysis based on preoperative clinical risk. Plast. Reconstr. Surg..

[14] Woerdeman L.A.E., Hage J.J., Hofland M.M.I., Rutgers E.J.T. (2007). A prospective assessment of surgical risk factors in 400 cases of skin-sparing mastectomy and immediate breast reconstruction with implants to establish selection criteria. Plast. Reconstr. Surg..

[15] Alderman A., Gutowski K., Ahuja A., Gray D. (2014). ASPS clinical practice guideline summary on breast reconstruction with expanders and implants. Plast. Reconstr. Surg..

[16] Doyle S.L., Lysaght J., Reynolds J.V. (2010). Obesity and post-operative complications in patients undergoing non-bariatric surgery. Obes. Rev. Off. J. Int. Assoc. Study Obes..

[17] Haslam D.W., James W.P.T. (2005). Obesity. Lancet.

[18] Kopelman P.G. (2000). Obesity as a medical problem. Nature.

[19] Gupta V., Winocour J., Rodriguez-Feo C., Bamba R., Shack R.B., Grotting J.C., Higdon K.K. (2016). Safety of Aesthetic Surgery in the Overweight Patient: Analysis of 127,961 Patients. Aesthetic Surg. J..

[20] Nguyen K.T., Hanwright P.J., Smetona J.T., Hirsch E.M., Seth A.K., Kim J.Y.S. (2014). Body mass index as a continuous predictor of outcomes after expander-implant breast reconstruction. Ann. Plast. Surg..

[21] Fischer J.P., Nelson J.A., Kovach S.J., Serletti J.M., Wu L.C., Kanchwala S. (2013). Impact of obesity on outcomes in breast reconstruction: Analysis of 15,937 patients from the ACS-NSQIP datasets. J. Am. Coll. Surg..

[22] Srinivasa D.R., Clemens M.W., Qi J., Hamill J.B., Kim H.M., Pusic A.L., Wilkins E.G., Butler C.E., Garvey P.B. (2020). Obesity and Breast Reconstruction: Complications and Patient-Reported Outcomes in a Multicenter, Prospective Study. Plast. Reconstr. Surg..

[23] Viscardi J.A., Oranges C.M., Schaefer D.J., Kalbermatten D.F. (2021). Reduction Mammoplasty: A Ten-Year Retrospective Review of the Omega Resection Pattern Technique. J. Clin. Med..

[24] World Health Organization (2021). “WHO/Europe|Nutrition—Body Mass Index—BMI,” World Health Organization. https://www.euro.who.int/en/health-topics/disease-prevention/nutrition/a-healthy-lifestyle/body-mass-index-bmi.

[25] Hanwright P.J., Davila A.A., Hirsch E.M., Khan S.A., Fine N.A., Bilimoria K.Y., Kim J.Y.S. (2013). The differential effect of BMI on prosthetic versus autogenous breast reconstruction: A multivariate analysis of 12,986 patients. Breast.

[26] Gabriel A., Sigalove S., Sigalove N.M., Storm-Dickerson T.L., Pope N., Rice J., Maxwell G.P. (2019). Effect of Body Mass Index on Outcomes after Prepectoral Breast Reconstruction. Plast. Reconstr. Surg..

[27] Gabriel A., Sigalove S., Storm-Dickerson T.L., Sigalove N.M., Pope N., Rice J., Maxwell G.P. (2020). Dual-Plane versus Prepectoral Breast Reconstruction in High-Body Mass Index Patients. Plast. Reconstr. Surg..

[28] Hubert H.B., Feinleib M., McNamara P.M., Castelli W.P. (1983). Obesity as an independent risk factor for cardiovascular disease: A 26-year follow-up of participants in the Framingham Heart Study. Circulation.

[29] Nguyen N.T., Magno C.P., Lane K.T., Hinojosa M.W., Lane J.S. (2008). Association of Hypertension, Diabetes, Dyslipidemia, and Metabolic Syndrome with Obesity: Findings from the National Health and Nutrition Examination Survey, 1999 to 2004. J. Am. Coll. Surg..

[30] Hart A., Funderburk C.D., Chu C.K., Pinell-White X., Halgopian T., Manning-Geist B., Carlson G., Losken A. (2017). The Impact of Diabetes Mellitus on Wound Healing in Breast Reconstruction. Ann. Plast. Surg..

[31] Ottesen T.D., Malpani R., Galivanche A.R., Zogg C.K., Varthi A.G., Grauer J.N. (2020). Underweight patients are at just as much risk as super morbidly obese patients when undergoing anterior cervical spine surgery. Spine J..

[32] Shida A., Ida M., Ueda M., Kirita T., Kawaguchi M. (2021). Preoperative underweight is associated with adverse postoperative events in patients undergoing microvascular reconstruction surgery for oral and maxillofacial cancer. Int. J. Oral Maxillofac. Surg..

[33] Weichman K.E., Broer P.N., Thanik V.D., Wilson S.C., Tanna N., Levine J.P., Choi M., Karp N.S., Hazen A. (2015). Patient-reported satisfaction and quality of life following breast reconstruction in thin patients: A comparison between microsurgical and prosthetic implant recipients. Plast. Reconstr. Surg..

[34] Yang C.-E., Chung S.W., Lee D.W., Lew D.H., Song S.Y. (2018). Evaluation of the Relationship Between Flap Tension and Tissue Perfusion in Implant-Based Breast Reconstruction Using Laser-Assisted Indocyanine Green Angiography. Ann. Surg. Oncol..

[35] Gabriel A., Maxwell G.P. (2017). Prepectoral Breast Reconstruction in Challenging Patients. Plast. Reconstr. Surg..

